# Circulating Senescent T Cells Are Linked to Systemic Inflammation and Lesion Size During Human Cutaneous Leishmaniasis

**DOI:** 10.3389/fimmu.2018.03001

**Published:** 2019-01-04

**Authors:** Luciana P. Covre, Régia F. Martins, Oliver P. Devine, Emma S. Chambers, Milica Vukmanovic-Stejic, Juliana A. Silva, Reynaldo Dietze, Rodrigo R. Rodrigues, Herbert L. de Matos Guedes, Aloísio Falqueto, Arne N. Akbar, Daniel C. O. Gomes

**Affiliations:** ^1^Núcleo de Doenças Infecciosas, Universidade Federal do Espírito Santo, Vitória, Brazil; ^2^Division of Infection and Immunity, University College London, London, United Kingdom; ^3^Saúde Global e Medicina Tropical, Instituto de Higiene e Medicina Tropical, Universidade Nova de Lisboa, Lisbon, Portugal; ^4^Instituto de Biofísica Carlos Chagas Filho, Universidade Federal do Rio de Janeiro, Rio de Janeiro, Brazil; ^5^Departamento de Medicina Social, Universidade Federal do Espírito Santo, Vitória, Brazil; ^6^Núcleo de Biotecnologia, Universidade Federal do Espírito Santo, Vitória, Brazil

**Keywords:** cutaneous leishmaniasis, immunosenescence, inflammation, SASP-analogous, *L. braziliensis*, T cells

## Abstract

*Leishmania (Viannia) braziliensis* induces American tegumentary leishmaniasis that ranges in severity from the milder form, cutaneous (CL) to severe disseminated cutaneous leishmaniasis. Patients with CL develop a cell-mediated Th1 immune response accompanied by production of inflammatory cytokines, which contribute to parasite control and pathogenesis of disease. Here, we describe the accumulation of circulating T cells with multiple features of telomere dependent-senescence including elevated expression of CD57, KLRG-1, and γH2AX that have short telomeres and low hTERT expression during cutaneous *L. braziliensis* infection. This expanded population of T cells was found within the CD45RA^+^CD27^−^ (EMRA) subset and produced high levels of inflammatory cytokines, analogous to the senescence-associated secretory profile (SASP) that has been described in senescent non-lymphoid cells. There was a significant correlation between the accumulation of these cells and the extent of systemic inflammation, suggesting that they are involved in the inflammatory response in this disease. Furthermore, these cells expressed high level of the skin homing receptor CLA and there was a highly significant correlation between the number of these cells in the circulation and the size of the *Leishmania*-induced lesions in the skin. Collectively our results suggest that extensive activation during the early stages of leishmaniasis drives the senescence of T cells with the propensity to home to the skin. The senescence-related inflammatory cytokine secretion by these cells may control the infection but also contribute to the immunopathology in the disease.

## Introduction

Parasites belonging to the *Leishmania* genus are among of the most diverse human pathogens in terms of both geographical distribution and clinical manifestations. Worldwide, 350 million people are at risk of acquiring the disease, 1.5–2 million new cases of *Leishmania* occur each year, and leishmaniasis causes 70,000 deaths per year in 88 tropical and subtropical countries. The disease ranges from a localized cutaneous to a fatal visceral form, depending on parasite species and host immunity (1).

Parasites from the *Viannia* subgenus that include *Leishmania* (*Viannia*) *braziliensis* are the most widespread species in the Americas, causing cutaneous leishmaniasis (CL) (1). The development of cutaneous lesions during CL is prevalent in more than 90% of cases and is characterized by an intense local cell-mediated Th1 immune response and production of inflammatory cytokines, which contribute to parasite control and tissue damage (2, 3). In addition, increased production of pro-inflammatory mediators, such as TNF-α, C-reactive protein (CRP), and adenosine deaminase (ADA) are observed in patients infected with *Leishmania* and may play an important role in the pathogenesis of disease (4, 5).

Immunosenescence is a term used to define the physiological decline in immune functions associated with the impaired ability of the host to mount an effective immune responses (6). In humans, immunosenescence naturally occurs with aging or is driven by chronic inflammatory conditions, inducing multiple phenotypic and dysfunctional characteristics in the T-cell pool (7).

Senescent T cells have been described in human CD4^+^ and CD8^+^ populations (8–10). These cells exhibit low proliferative potential after activation, short telomeres and low telomerase activity, elevated reactive oxygen species (ROS) production, constitutive p38 MAP kinase activation, expression of DNA damage response machinery and increased cyclin-dependent kinase inhibitor p16^INK4a^ expression (11). The senescent T cells express the CD45RA^+^CD27^−^ EMRA phenotype and have preferential homing capacity for peripheral tissues (12). Furthermore, these senescent T cells, secrete high levels of pro-inflammatory cytokines that has been suggested to be a manifestation of the senescence associated secretory phenotype (SASP), as described previously in non-lymphoid cells (10, 13).

Although T cell dysfunctions are linked to increased replication and survival in parasite diseases (14, 15) there is no data associating T cell senescence and *Leishmania* infection so far. Here, we demonstrate that the intense inflammatory cytokine secretion that has previously been shown to occur in this disease is linked to the accumulation of senescent T cells with a SASP-like functional profile. Furthermore, these circulating cells have skin homing potential and correlate numerically with leishmanial-related skin lesion size.

Collectively our data offer a re-interpretation of the immune events that occur during leishmaniasis by demonstrating that the intensive proliferative drive to T cells induces T senescence and associated pro-inflammatory cytokine secretion that contributes to parasite control and also immunopathology during cutaneous leishmaniasis.

## Materials and Methods

### Study Subjects

Peripheral blood from 17 untreated cutaneous leishmaniasis (CL) patients attendant at Hospital Universitário Cassiano Antônio de Morais, Universidade Federal do Espirito Santo–Brazil were used in this study. The diagnosis of CL was determined by clinical and laboratory criteria. All patients in this study tested positive for the PCR/restriction fragment length polymorphism for *L. braziliensis* and reported no prior *Leishmania* infections or treatment (Supplementary Figure 1). The control group consisted of 15 healthy age matched individuals (HC) from non-endemic areas, without treatment history. All study participants consented to take part and were serologically negative for HIV, HBV, and HCV infection. They also had no history of chemotherapy, radiotherapy or treatment with immunosuppressive medications within the last 6 months. All volunteers and patients provided written informed consent, and study procedures were performed in accordance with the principles of the Declaration of Helsinki. The study was registered at HUCAM ethical committee under referential number 735.274.

### PBMC and T Cells Isolation

PBMCs from CL and HC were isolated by centrifugation of heparinized whole blood through a Ficoll-Hypaque gradient (GE Healthcare, Uppsala, Sweden). Cryopreserved cells from both controls and patients were thawed in RPMI complete medium supplemented with 10% of fetal calf serum. Viability and recovery were measured using trypan blue dye exclusion in haemocytometer. The CD4^+^ and CD8^+^ T cell isolations were performed by negative selection using MACS cell separation (Milteny Biotec) according to the manufacturer's protocol.

### Flow Cytometric Analysis

Flow cytometric analysis was carried out using the following reagents: Live/dead UV Zombie, KLRG1 APC (2F1), CD4 Brilliant Violet 711 (OKT-4), CD28 Brilliant Violet 510 (CD28.2) and CD45RA PECy7 (HI100) all from Biolegend. CD28 Brilliant Violet 786 (U28), CD3 PECF594 (UCHT1), CD8 APC-H7 (SK1), CD57 Alexa Fluor 421 (NK-1), and human cutaneous lymphocyte antigen (CLA) FITC (HECA-452) from BD Biosciences. Cell suspensions were incubated with antibody solutions for 30 min at 4°C for extracellular staining. Intracellular staining for Ki67 (clone B56, BD Bioscience) was performed with Foxp3 Staining Buffer Set (Miltenyi Biotec, Bisley, UK). Cytokine and anti-hTERT (rabbit IgG—Abcam) staining were performed using Fix and Perm Cell Permeabilization Kit (Invitrogen, Paisley, UK) according to the manufacturer's protocol. Samples were processed at Fortessa X-20 cytometer (BD Biosciences) and analyzed using FlowJo software (Treestar). Isotype control staining and fluorescence-minus-one controls were used to set the quadrants.

### Phospho-Flowcytometry

The levels of p38 (pT180/pY182) Alexa Fluor 488 and γH2AX (pSer139) Alexa Fluor 647 (B56), all from BD Biosciences were analyzed *ex vivo* in PBMCs fixed with warm Cytofix Buffer (BD Biosciences) at 37°C for 10 min. Cells were permeabilized with ice-cold Perm Buffer III (BD Biosciences) at 4°C for 30 min and incubated with the respective antibodies for 30 min at room temperature.

### Cytokine Determination

Cytokine production was determined in the supernatant of PBMC unstimulated or stimulated samples with 10 μg/ml of *L. braziliensis* promastigote antigens (LbAg) or 0.5 μg/mL plate-coated anti-CD3 (OKT3) (Biolegend) in the presence of 5 ng/mL of rhIL-2 for 72 h. IFN-γ, TNF-α, Granzyme B, IL-10, and IL-4 levels were assayed by Cytokine Bead Array (CBA) (BD Biosciences) according to the manufacturer's protocol. Serum evaluation of IL-10, IFN-γ, TNF-α, and CRP levels were performed by ELISA assay according to the manufacturer's protocol (R&D systems).

### Proliferation Assay

Proliferation was performed by using PBMC unstimulated or stimulated with 0.5 μg/mL plate-coated anti-CD3 (OKT3) (Biolegend) in the presence of 5 ng/mL of rhIL-2 for 72 h followed of evaluation by intracellular staining for the cell cycle related nuclear antigen Ki67 by flow cytometry.

### Telomere Fluorescence *in situ* Hybridisation

CD4^+^ and CD8^+^ T cells were isolated (as above) from HC and CL samples and prepared on poly-L-lysine coated glass slides by cytocentrifugation (Cytospin, Thermo Scientific, USA). Cytological specimens were then dried and fixed in ethanol/acetone prior to freezing. Specimens were thawed prior to staining. Specimens were PFA fixed, dehydrated in cold ethanol prior to permeabilization and blocking with BSA. Slides were washed prior to further dehydration across an ethanol gradient and air dried prior to hybridization with the PNA probe (Panagene, TelC Cy3, #14 1224PL-01) for 2 h in the dark as described in (16). Slides were subsequently washed in formamide/SSC prior to mounting with Vectorshield/DAPI (Vector Laboratories, USA). Imaging was performed using a Leica SPE2 confocal microscope using LAS X version 3.3.0 software (Leica Microsystem, Wetzlar, Germany). The images corresponded to a full z stack of CD4 or CD8 T cells were analyzed using ImageJ software.

### *t*-SNE Analysis

Unbiased representations of multi-parameter flow cytometry data were generated using the *t*-distributed stochastic neighbor embedding (*t*-SNE) algorithm. The R package “Rtsne” available on CRAN (github.com/jkrijthe/Rtsne) was used to perform the Barnes Hut implementation of *t*-SNE on flow cytometry data. FlowJo software was used to export events of interest (in fcs format) for each sample. After using the Bioconductor “flowCore” R package to import.fcs file data and the Logicle transform to scale the data similarly to that displayed in FlowJo, 10.000 events from each samples analyzed in parallel were merged and the relevant fluorescent parameters were used. *t*-SNE is a non-linear dimensionality reduction method that optimally places cells with similar expression levels near to each other and cells with dissimilar expression levels further apart.

### Statistics

GraphPad Prism was used to perform statistical analysis. KS normality test was prior performed and the statistical significance was evaluated using Student *t*-test for paired samples or repeated-measures ANOVA with Tukey correction used for *post-hoc* testing. A Mann-Whitney test was performed for all continuous, non-parametric variables. Differences were considered significant when *P* was < 0.05.

## Results

### T Cells From Cutaneous Leishmaniasis Patients Exhibit Characteristics of Senescence

**Figure 1:**
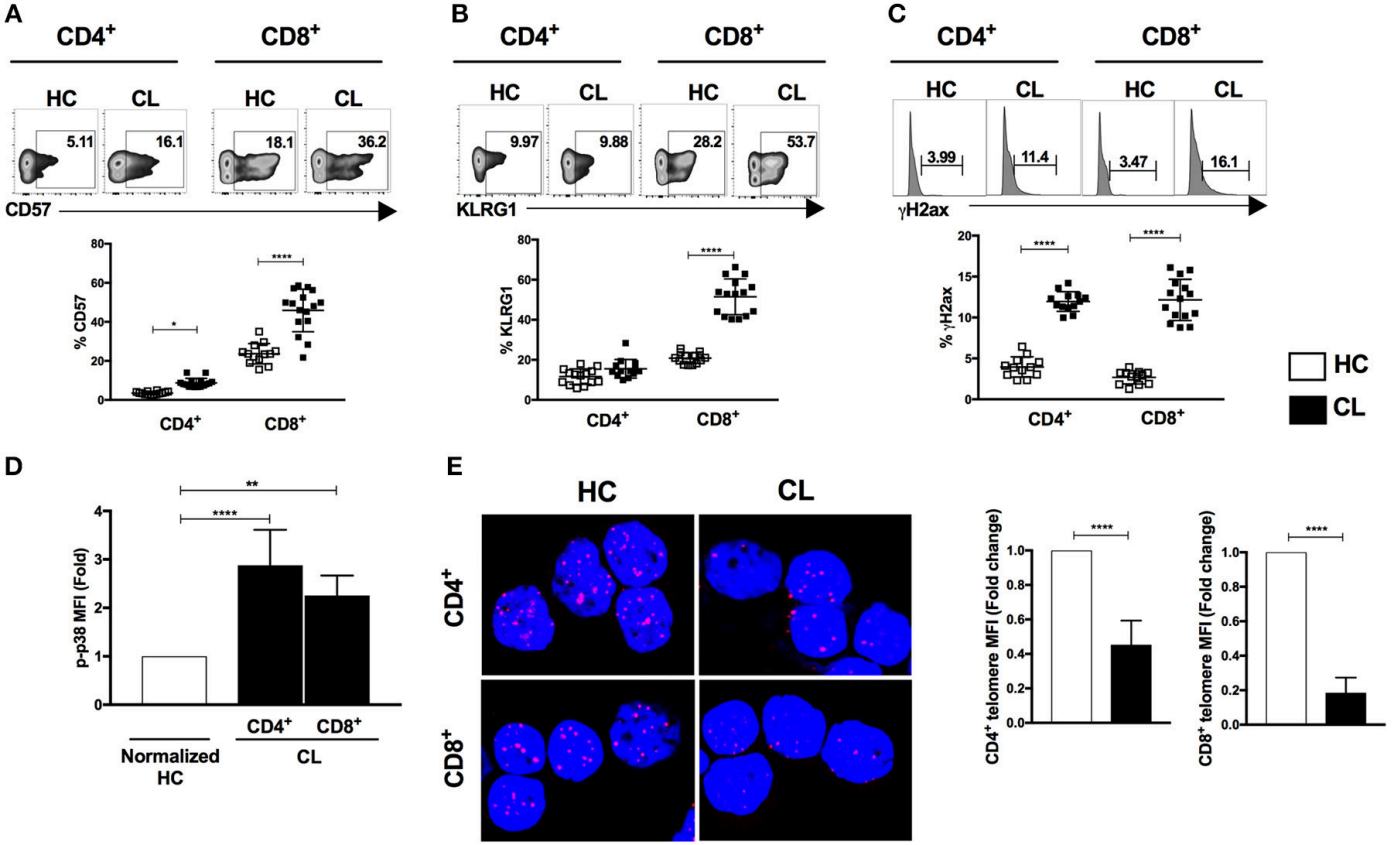
Characterization of senescent markers in T cells during cutaneous leishmaniasis. CD4^+^ and CD8^+^ T cells isolated from healthy controls (HC) (*n* = 14) and patients with active cutaneous leishmaniasis patients (CL) (*n* = 16) were stained for CD57, KLRG1, phosphorylated histone H2AX (γH2AX) or phosphorylated p38 protein and analyzed by flow cytometry. Representative cytometry plots and cumulative data of CD57 **(A)**, KLRG1 **(B)**, γH2AX- expressing cells **(C)**, fold change of fluorescence intensity of phospho-p38 protein **(D)**, telomere FISH image in purified CD4^+^ and CD8^+^ T cells hybridized with telomere probe and fold change of quantitative fluorescence intensity levels normalized with HC group **(E)**. The graphs show the mean ± SEM. *P*-values were calculated using repeated-measures ANOVA with the Tukey correction used for *post-hoc* testing. ^*^p < 0.05, ^**^p < 0.01, ^****^p < 0.0001.

Senescent T cells demonstrate both intra- and extra- cellular phenotypic changes as well as functional defects often implicated in the susceptibility of host immunity. We observed increased frequencies of the cells surface senescence associated markers CD57 in CD4^+^ and CD8^+^ T cells (Figure 1A) and the inhibitory killer cell lectin-like receptor G1 (KLRG1) in CD8^+^ T cells in CL patients compared to controls (Figure 1B). T cells from patients also expressed more of the DNA damage-related protein γH2AX (Figure 1C) and exhibited elevated phosphorylation of p38 protein that was significantly higher in CD4^+^ and CD8^+^ T cells than controls (Figure 1D). In addition, confocal microscopy analysis demonstrated shorter telomeres in both CD4^+^ and CD8^+^T cells that were significantly shorter than observed in the HC control group (Figure 1E). Therefore, T cells that exhibit multiple characteristics of senescence accumulate in patients with CL.

### Senescent T Cells From Cutaneous Leishmaniasis Patients Are Found Within the EMRA Population

**Figure 2:**
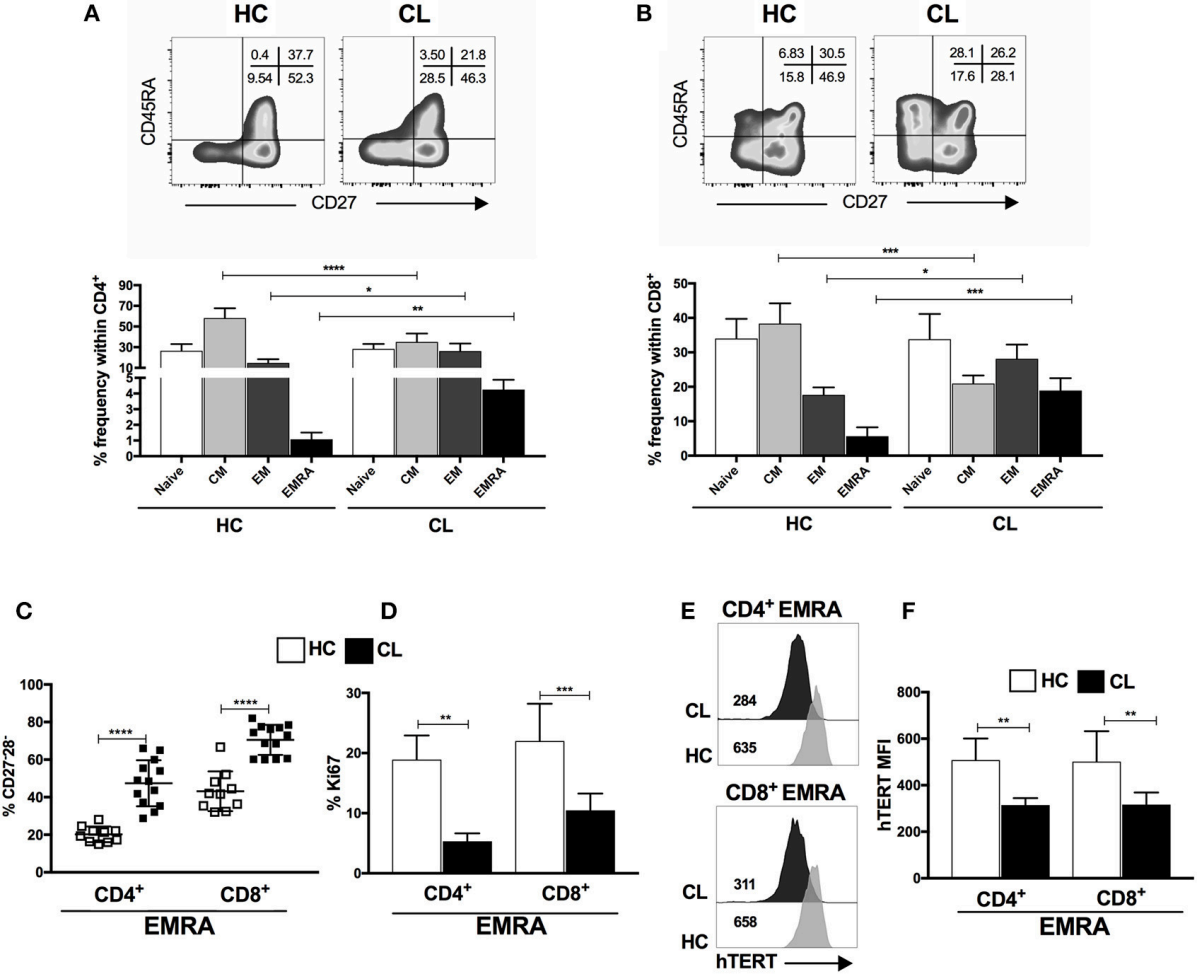
End-stage memory T cells (EMRA) accumulate during the CL and displays functional characteristics of senescence. Representative plots and cumulative data of percentage of CD4^+^ **(A)** and CD8^+^ **(B)** T cells subsets isolated from HC (*n* = 12) or CL (*n* = 14) and characterized by expressing CD45RA and CD27 markers (naive-CD45RA^+^ CD27^+^; CM-central memory, CD45RA^−^ CD27^+^; EM-effector memory, CD45RA^−^CD27^−^; and EMRA-effector memory T cells that re-express CD45RA, CD45RA^+^ CD27^−^). Frequencies of CD27 and CD28 co-stimulatory molecules loss within CD4^+^ and CD8^+^ EMRA subset **(C)**. Proliferative capacity evaluated by Ki67 staining **(D)** and telomerase reverse transcriptase (hTERT) MFI **(E)** of CD4^+^ and CD8^+^ EMRA subset **(F)** from HC or CL stimulated with 0.5μg/mL of anti-CD3 for 72 h. The graphs show the mean ± SEM. *P*-values were calculated using repeated measures ANOVA with the Tukey correction used for *post-hoc* testing. ^*^p < 0.05, ^**^ *p* < 0.01, ^***^p < 0.001, ^****^p < 0.0001.

Both CD8^+^ and CD4^+^ T cells can be subdivided into four populations on the basis of their relative surface expression of CD27 and CD45RA molecules. This can define naive (CD45RA^+^CD27^+^), central memory (CM; CD45RA^−^CD27^+^), effector memory (EM; CD45RA^−^CD27^−^), and effector memory T cells that re-express CD45RA (EMRA; CD45RA^+^CD27^−^) that contain the majority of senescent T cells (8). CL patients possess increased frequencies of effector memory (EM) and EMRA and decreased central memory (CM) populations in both CD4^+^ and CD8^+^ T cell subsets (Figures 2A,B). No difference in total circulating white cell count was observed between healthy controls and CL patients (Supplementary Figure 1) indicating a proportional increase of these cells in the circulating CD4^+^ and CD8^+^ subsets.

Chronic infection and continuous antigen-specific stimulation of T cells induces loss of co-stimulatory receptors, such as CD28 and CD27 and replicative capacity resulting from telomere shortening and decreased activity of the enzyme telomerase. The CL-EMRA T cell subset within both CD4^+^ and CD8^+^ compartments have lost CD27 and CD28 coexpression (Figure 2C) and demonstrated diminished proliferative capacity (Figure 2D) that was also observed in both CM and EF memory subsets (Supplementary Figure 2) and identified by Ki67 staining, after polyclonal stimulation *in vitro*. We analyzed the expression of catalytic telomerase component (hTERT) within CD4^+^ and CD8^+^ EMRA from both groups and found that CL patients exhibit decreased expression of hTERT compared to controls (Figures 2E,F). This observation was extended to CM and EM memory subsets of both compartments (CD4^+^ and CD8^+^) in response to activation (data no shown). Therefore, the EMRA subset that accumulates during CL exhibits phenotypic as well as functional characteristics of senescence.

### T Cells From Cutaneous Leishmaniasis Patients Exhibit a Pro-Inflammatory Functional Phenotype

**Figure 3:**
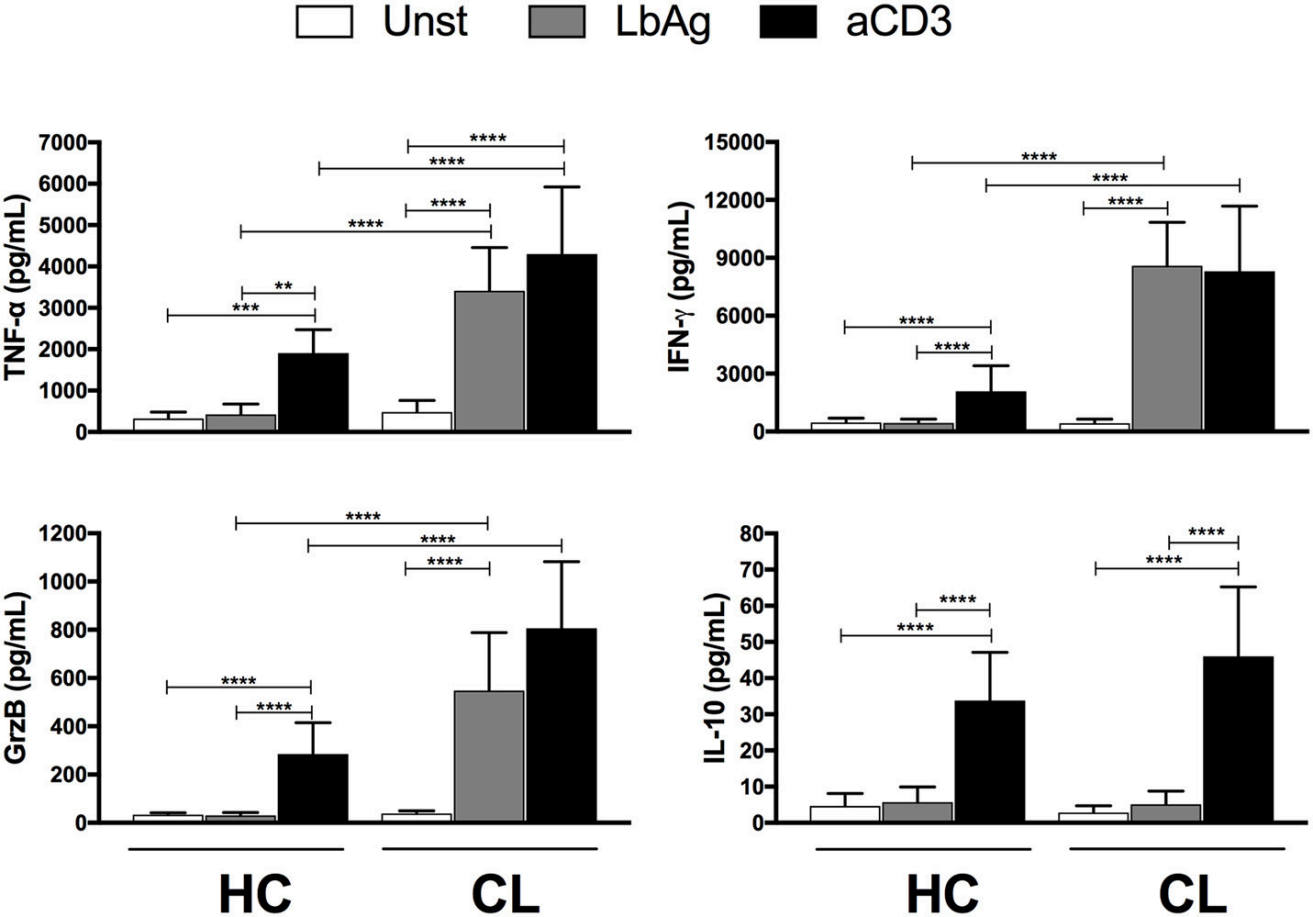
Cytokines production by activated cells. PBMC from HC (*n* = 13) or CL (*n* = 14) groups were cultured with 10 μg/ml of *L. braziliensis* promastigote antigens (LbAg) or 0.5 μg/mL of anti-CD3 for 72 h. Production of IFN-γ, TNF-α, Granzyme B, and IL-10 were determined in the culture supernatants by CBA. The graphs show the mean ± SEM. *P*-values were calculated using repeated measures ANOVA with the Tukey correction used for *post-hoc* testing. ^**^p < 0.01, ^***^p < 0.001, ^****^p < 0.0001.

The abundant capacity to secrete pro-inflammatory mediators including cytokines has been associated with aging and chronic infections. Compared to controls, the PBMC from CL patients secreted significantly higher levels of IFN-γ, TNF-α, and Granzyme B after anti-CD3 stimulation (Figure 3). In addition, *L. braziliensis* antigen (LbAg) stimulation induced high levels of these mediators in CL patients but not in the healthy controls, suggesting that there is an accumulation of parasite specific T cells with a secretory profile in these individuals. While IL-10 was induced after anti-CD3 stimulation of patients and controls, LbAg stimulation did not induce this inhibitory cytokine in CL patients indicating an imbalance of pro-inflammatory vs. anti-inflammatory cytokines in response to specific antigen stimulation.

**Figure 4:**
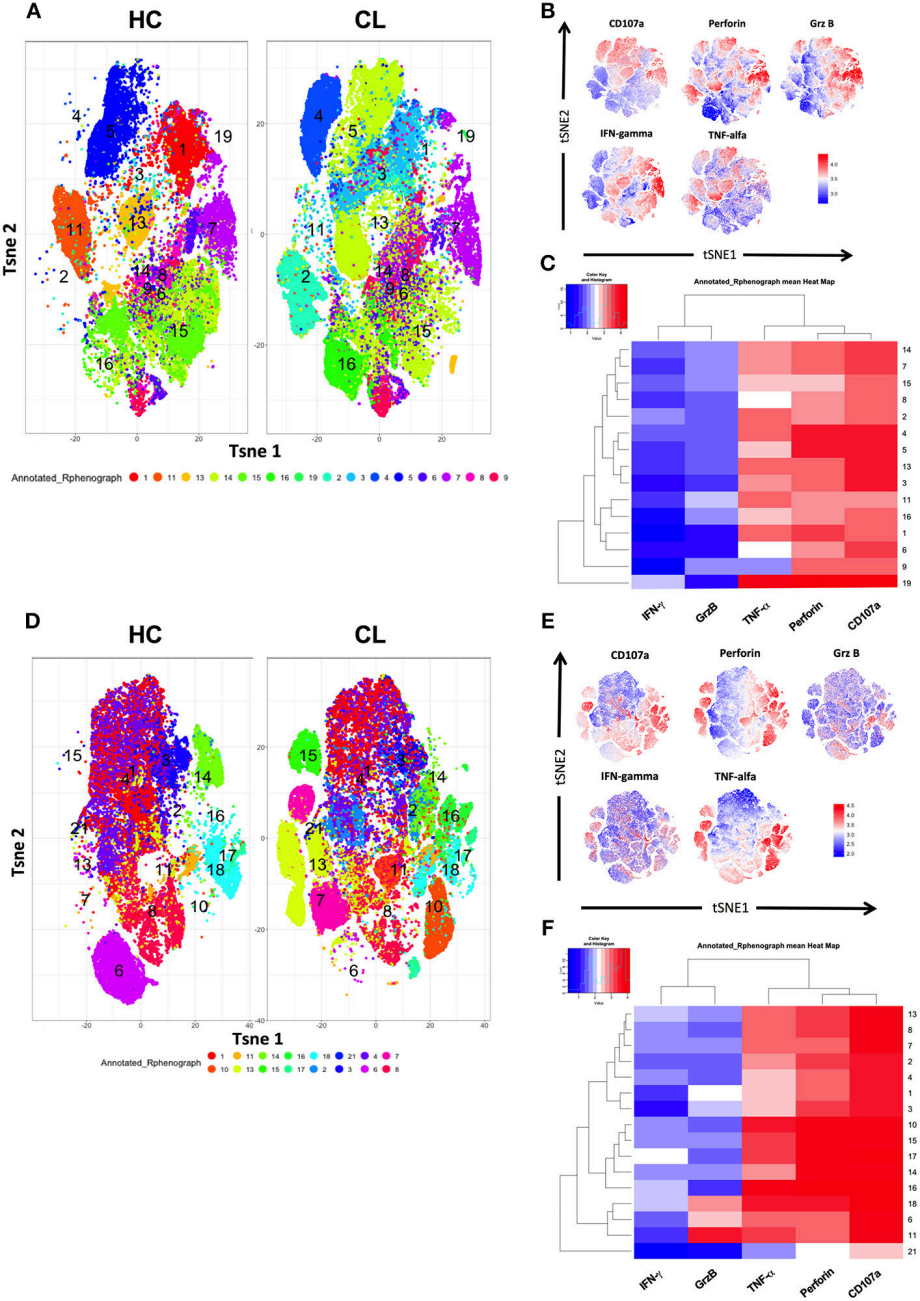
Pro-inflammatory cytokines and cytotoxic granules are highly diverse across CD4^+^ and CD8^+^ T cells from HC and CL patients. PBMC from healthy donors (HC) (*n* = 14) and cutaneous leishmaniasis patients (CL) (*n* = 16) were stimulated with 0.5 μg/mL of anti-CD3 for 24 h and stained for surface and intra-cellular markers for cytometry analysis. *t*-SNE was performed gating on CD4^+^ and CD8^+^ T cells, which are identified general differences in functional markers expression between both compartments from HC and CL groups and evidenced by the numbered and colored clusters **(A,D)**. The expression of granzyme B, IFN-γ, TNF-α, CD27, CD45RA, perforin, and CD107a were evaluated separately on live CD4^+^ and CD8^+^ T cells **(B,E)**. Heatmap showing hierarchical clusters and functional markers expression levels of CD4^+^ and CD8^+^ T cells from HC and CL patients **(C,F)**. Normalized protein expression levels were represented in red for high expression, whereas blue represents low expression (cold-to-hot heat map).

To investigate the pro-inflammatory cytokine secretion in more detail we used the *t*-Distribution Stochastic Neighbor Embedding (*t*-SNE) algorithm, which projects high dimensional data into two dimensional space (*t*-SNE1 and *t*-SNE2) through performing repeated pairwise comparison of randomly selected cellular phenotypes based on their marker expression—ultimately clustering closely related cells. This allowed us to characterize the phenotypic diversity identified by specific clusters associated with granular contents (Granzyme B, Perforin, and CD107a) and cytokines (IFN-γ, TNF-α) on CD4^+^ (Figures 4A,B) and CD8^+^ T cells (Figures 4D,E) obtained from healthy controls and CL patients. The relative low expression of pro- inflammatory mediators in HC were combined with a strong expression of them in both T cell compartments of CL patients. This indicates the differential composition of functional markers with the predominance of TNF-α, CD107a, and perforin associated to CL-CD4^+^ (2, 4, 14, and 19) and CD8^+^ (7, 8, 10, 16, 13, and 15) clusters, but not in controls, as presented in the heat map (Figures 4C,F).

**Figure 5:**
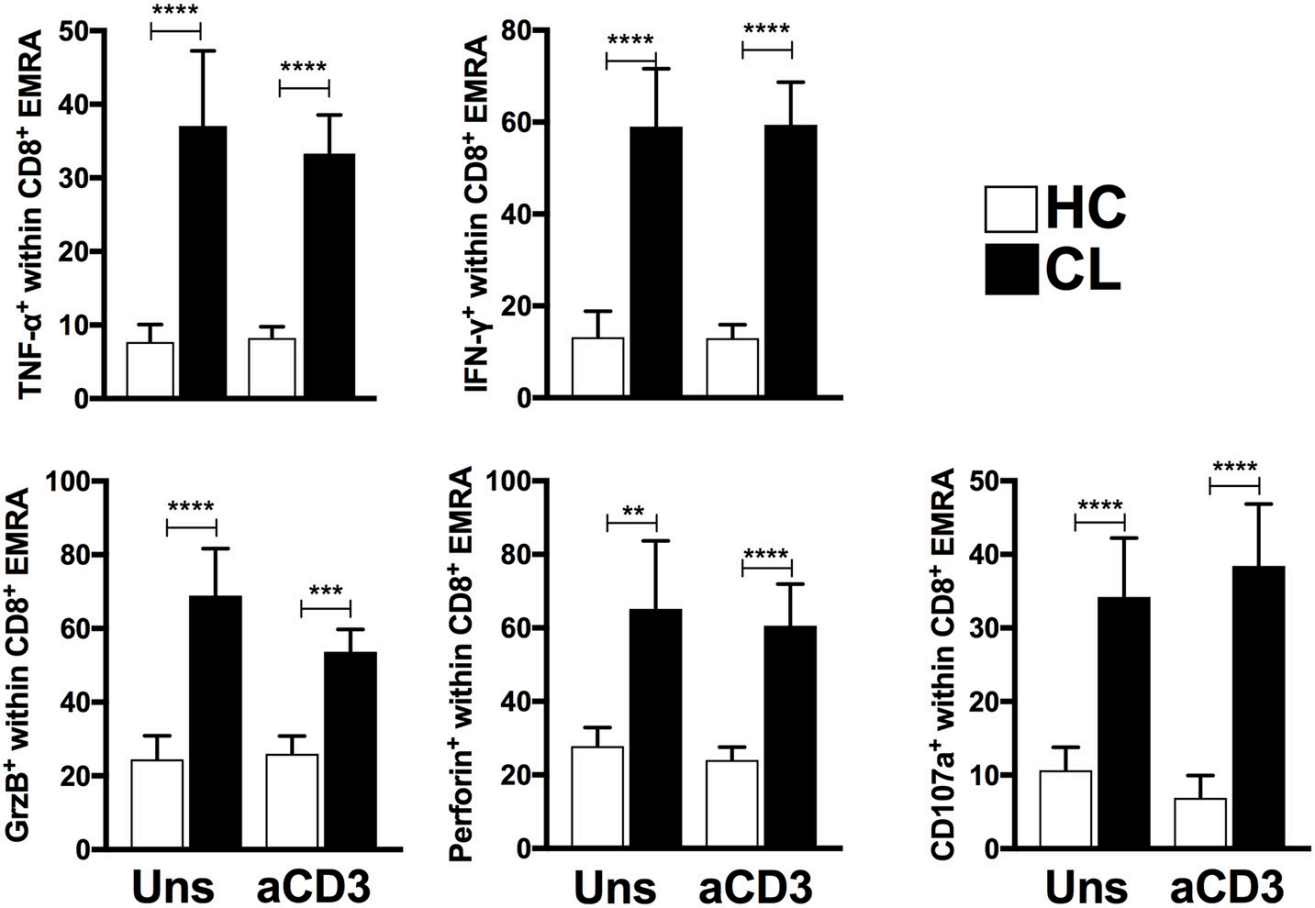
EMRA subsets from CL patients present evident senescent-associated secretory phenotype. Cumulative data of perforin, CD107a, TNF-α, IFN-γ, and Granzyme B-producing CD8^+^-EMRA frequencies from HC (*n* = 11) and CL (*n* = 13) unstimulated or stimulated with 0.5 μg/mL of anti-CD3 stimulation for 24 h. The graphs show the mean ± SEM. *P*-values were calculated using repeated measures ANOVA with the Tukey correction used for *post-hoc* testing. ^**^p < 0.01, ^***^p < 0.001, ^****^p < 0.0001.

To investigate this further we examined the secretory phenotype of cytokine-producing T cells within CL patient and HC by flow cytometric analysis using the expression of senescence surface markers CD45RA and CD27. *Ex vivo* and polyclonal stimulation analyses demonstrated increased frequencies of senescent T cells subset (EMRA) within both CD8^+^ (Figure 5) and CD4^+^ T cells (data not shown) from CL patients that produce IFN-γ, TNF-α, Granzyme B, Perforin, and CD107a, supporting the pro-inflammatory secretory profile of these populations.

### CL Patients Have Increased Systemic Inflammation That Correlates With the Accumulation of Senescent (EMRA) and Effector Memory T Cells

**Figure 6:**
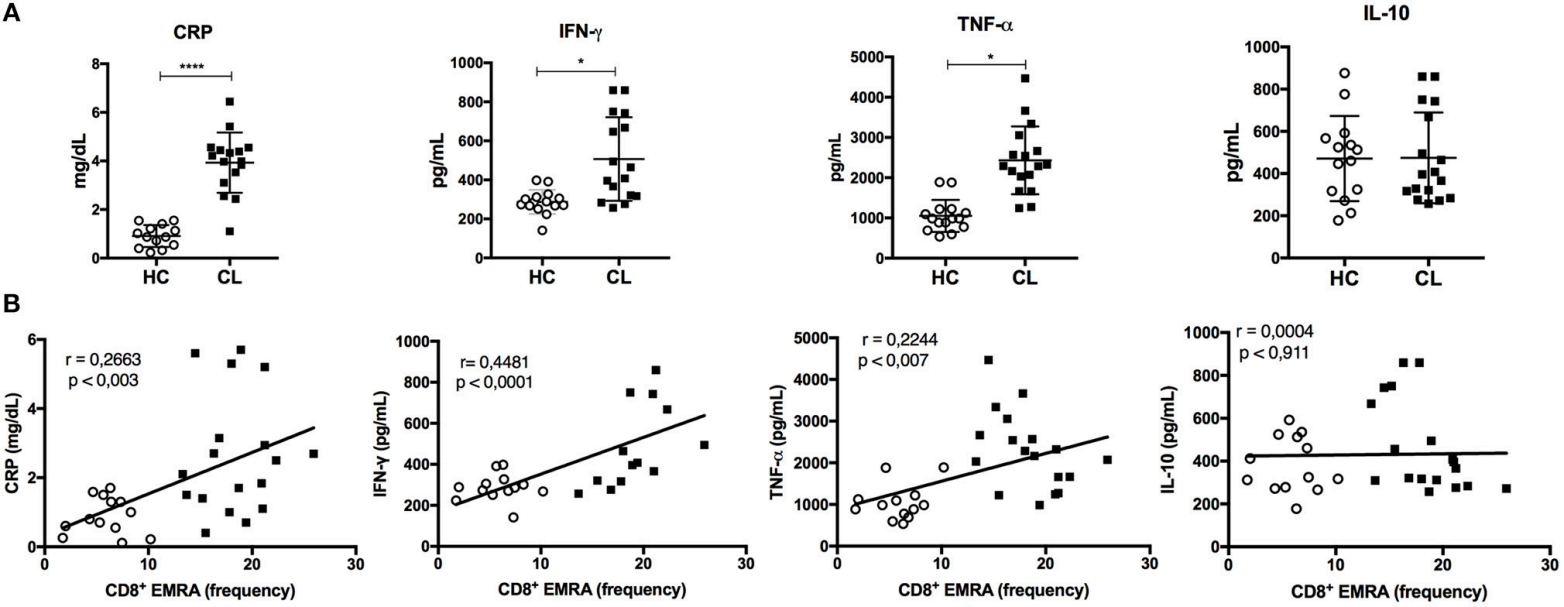
Inflammatory profile is exhibited in the serum of CL patients and correlates with EMRA frequency. Serological analysis of C-reative protein (CRP), IFN-γ, TNF-α, and IL-10 levels in CL patients (*n* = 16) and healthy control group (HC) (*n* = 15) **(A)**. Correlation between cytokines or CRP levels and frequency of CD8^+^ EMRA T cells in HC (○) or CL patients (■) were tested using Pearson's correlation test **(B)**. For all tests, a ^*^p < 0.05, ^****^p < 0.0001 were considered statistically significant.

Systemic production of pro-inflammatory mediators is linked to the development of phenotypic and dysfunctional senescence features of T cells. Thus, we performed a serological analysis for some common immune mediators in both HC and CL groups. CL patients had increased systemic levels of C-reactive protein (CRP), IFN-γ, and TNF-α (Figure 6A), which correlated significantly with the accumulation of senescent CD8^+^ (Figure 6B) and CD4^+^ (Supplementary Figure 3) T cell subsets. Moreover, CRP levels correlated positively with the accumulation of EF memory T cell within both CD4^+^ and CD8^+^, but not naïve or central memory subsets (Supplementary Figure 4). There was no correlation between the level of IL-10 and EMRA T cells further suggesting an imbalance between pro and anti-inflammatory cytokines in CL patients. There was also no correlation between the levels of these cytokines and the CM or EM memory T cell subsets (data not shown).

### Circulating Senescent (EMRA) T Cells Correlate With Lesion Size and Propensity for Skin Homing After *L. braziliensis* Antigen Stimulation

**Figure 7:**
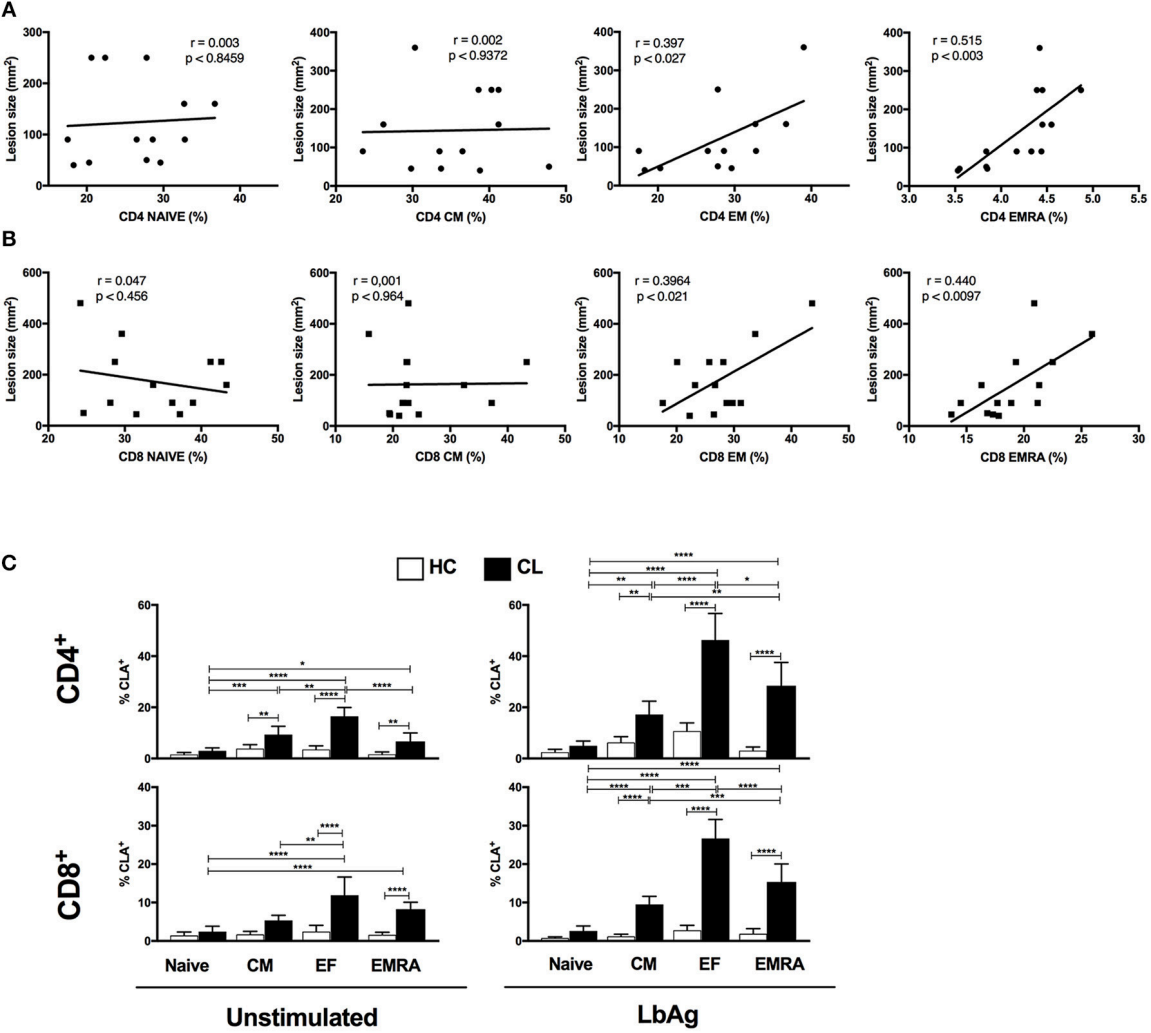
CL- EMRA subset correlates with lesion size and up regulate the skin homing receptor cutaneous leucocyte-associated antigen (CLA). Pearson's correlation test between frequencies of CD4^+^ and CD8^+^ T subsets (Naïve, CM, EF, and EMRA) and lesions size (mm^2^) of CL patients (*n* = 14) **(A,B)**. Cumulative data of CLA- expressing T cell subsets from HC and CL **(C)** in response to 10 μg/ml of *L. braziliensis* promastigote antigen (LbAg) stimulation for 72 h and analyzed by flow cytometry. The graphs show the mean ± SEM. *P*-values were calculated using repeated measures ANOVA with the Tukey correction used for *post-hoc* testing. ^*^p < 0.05, ^**^p < 0.01, ^***^p < 0.001, ^****^p < 0.0001.

As senescent T cells that accumulated in CL patients were pro-inflammatory, especially after stimulation with *L. braziliensis* antigens, we investigated if they were associated with the skin immunopathology that occurs during the infection. To do this we correlated the EMRA CD4^+^ and CD8^+^ T cell subsets with the size of the skin lesions in patients with cutaneous leishmaniasis (Figure 7A). We found a strong correlation between the EMRA CD4^+^ and moderate correlation between CD8^+^ T cell subsets cells with lesion size suggesting that these circulating cells contributed to skin immunosurveillance and immunopathology during the course of the disease. The same correlation was observed with EM subset from both compartments, supporting previous observations (17–19).

We therefore evaluated the ability of EMRA subsets to regulate the skin homing receptors cutaneous leucocyte-associated antigen (CLA) and chemokine receptor CCR4 before and after activation with LbAg. Polyclonal activation with anti-CD3 induced increased expression of CLA in both CL and HC groups (not shown), however EMRAs and EM from CL upregulated the CLA expression in response to *L. braziliensis* antigen recall (Figures 7B,C). Circulating CD4^+^ and CD8^+^ T cells showed higher frequencies of CLA compared to HC group (Supplementary Figure 5) confirming previous reports (18, 19). However, we extended previous observations by showing that LbAg induced high levels of CLA predominantly in EF and EMRA memory T cell subsets within both CD4^+^ and CD8^+^ compartments. These findings suggest that there is preferential induction of CLA on T cells from CL conferring increased ability to target skin lesion sites. No differences in CCR4 expression were observed between HC and CL groups in none of the conditions evaluated (data not shown).

## Discussion

Persistent antigenic stimulation can result in phenotypic characteristics and functional changes associated with senescence in human T cells. These include decreased responsiveness to TCR stimulation, diminished proliferative capacity, increased susceptibility to apoptosis and expression of immune inhibitory receptors (11, 20) that directly impede the host effort to mount an effective immunity against pathogens (21, 22).

In our experiments, both CD4^+^ and CD8^+^ T cells from CL patients exhibit increased expression of many senescence-related markers including CD57, KLRG1, p38 MAPK, and phosphorylation of histone protein H2AX. Although the increased expression of these markers has been described previously in aging and infection (23, 24) it was not clear if these cells also accumulated during parasitic infections. As previously reported and supported by our findings, circulating CD4^+^ and CD8^+^ T cells from CL patients demonstrated higher expression of CD57 as compared to healthy controls (25, 26), and this was also observed in T cells from the lesions of CL patients or during visceral leishmaniasis (27).

KLRG1 expression has been shown to impair microbicidal function of NK cells and CD8^+^ T cells (28, 29) and this may have an impact on host immunity during *Leishmania* infections. In addition, murine CD8^+^ T cells expressing KLRG1 downregulate its receptor under pro-inflammatory conditions, which is essential for developing peripheral and tissue-resident memory cells with increased proliferative capacity (30); this supports the limitation of the isolated use of KLRG1 as a senescent marker. However, in the present and previous studies (31), we have used KLRG1 expression in conjunction with other markers to define senescent T cells.

The phosphorylation of both p38MAPK and H2AX is induced in response to telomere shortening, which have been associated with proliferative defect in human T cells (32). This is related to minimal telomerase activity that in other situations can be restored with the transduction of hTERT resulting in telomere elongation and an increase in replicative life span after stimulation (8, 33). Consistent with these, both CD4^+^ and CD8^+^ T cells from CL patients show short telomeres and low expression of the catalytic telomerase component in memory subsets.

Similar to our findings, continued antigenic exposure drives the expansion and differentiation of both CD4^+^ and CD8^+^ T cells to an end-stage/senescent population that re-express CD45RA associated with the loss of CD27/CD28 co-stimulatory receptors (EMRA). As extensively demonstrated in HIV and CMV infections these populations exhibit decreased proliferative capacity, as demonstrated here and increased propensity to apoptosis and decreased TCR stimulation (20). Although we did not perform analyzes regarding propensity to apoptosis and decreased TCR stimulation that could be a limitation to the study, our previous observations we have demonstrated that T cells with the phenotype of senescent cells as described here do exhibit these characteristics (9, 34).

Cutaneous leishmaniasis caused by *L. braziliensis* is also are associated with potent Th1-type responses and chronic production of pro-inflammatory cytokines (2, 3). Although these responses are beneficial for the activation of leishmanicidal mechanisms and parasite control, the exaggerated inflammation contributes to the development of skin lesions and tissue destruction observed in human ML and CL patients (35, 36). He we show that the inflammatory T cells that accumulate in the circulation of infected individuals have characteristics of senescent cells and since they are highly secretory, they may have the equivalent of a senescence-associated secretory phenotype (SASP) (13). In addition, we consider that the extent of senescence may depend on the clinical type of leishmaniasis as well as during other parasitic infections (27, 37, 38). A key observation was that the stimulation with *Leishmania* antigens induced an imbalance in the pro vs. anti-inflammatory cytokine ratio compared with anti-CD3 activation of T cells from infected patients. In patients and murine models, the exacerbated production of IFN-γ and TNF-α, and the decreased production of IL-10 positively correlate with lesions size in CL and tissue damage in severe mucosal leishmaniasis (ML) (35, 36). Intralesional analysis of cytokine gene expression has shown high frequencies of granzyme, IL-2, IFN-γ, and TNF-α and enrichment of *Leishmania*-specific CD8^+^ T (19, 39). Moreover, patients treated with anti-inflammatory drugs show increased healing of lesions, supporting the deleterious role of inflammation (40, 41). High frequencies of cytotoxic cells were observed in lesions of patients with CL caused by *L. braziliensis* and correlated with inflammatory potential and cutaneous tissue damage. Moreover, the exaggerated inflammation correlates with increased tissue hyperplasia, granuloma development, T cells apoptosis and susceptibility to visceral leishmaniasis (19, 39, 42).

Increased inflammatory markers have been linked to a wide variety of adverse outcomes and chronic conditions, even as reciprocally impaired antigen-specific immune responses compromise protective capacities (43). According to our experiments, increased levels of pro-inflammatory mediators are observed systemically in patients with both CL and ML or induced *in vitro* after specific-antigen recall (5, 44, 45). Inflammatory products, such as TNF-α, IL-1β, CRP are potent inductor of phenotypic and dysfunctional senescence features of T cells accelerating the impairment of the immune effector function (7).

We now show that circulating senescent cells in infected individuals have increased potential to home to the skin as indicated by high CLA expression, extending previous reports that demonstrate the up regulation of this receptor in circulating and local effector memory T cells (17–19). CLA is a unique skin-homing receptor expressed by circulating memory T cells that infiltrate the skin which facilitates targeting of T cells to inflamed skin, which also has been associated with the pathogenesis or severity of many inflammatory skin diseases, such as leprosy, atopic dermatitis and psoriasis (46, 47). Furthermore, the activation of T cells from patients with *L. braziliensis* antigens *in vitro* further induced the expression of CLA but not CCR4 on the senescent T cells. During *Leishmania* infection skin homing receptors can be induced by proinflammatory mediators, such as IL-1 and TNF-α as consequence of tissue injury and systemic inflammation (40). CLA-positive cells can use both VLA-4/VCAM-1 and LFA-1/ICAM-1 for extravasation on skin surfaces (48), which could account the enrichment of T cells expressing this receptor in the lesion of cutaneous leishmaniasis patients (18, 49).

The observation of a positive correlation between the accumulation of circulating senescent T cells and lesion size provides a link between events in the blood and in the skin suggesting that the recruitment of these proinflammatory cells, resulting from increased CLA expression, may contribute to the skin pathology in CL.

The findings presented here demonstrate that senescent T cells with a unique functional profile are extensively induced by telomere dependent-senescence during cutaneous leishmaniasis. This study identifies the role of cellular senescence in regulating the function of parasite-specific T cells and demonstrates that these cells can also be associated with the immunopathology that occurs during acute infection.

## Author Contributions

LC, RM, OD, HdM, JS, and DG performed experiments. LC, DG, OD, and EC analyzed data. AF selected the patients. DG, AA, RD, and MV-S designed the project and discussed data. DG, AA, MV-S, RR, and EC wrote the manuscript the support of all other co-authors.

### Conflict of Interest Statement

The authors declare that the research was conducted in the absence of any commercial or financial relationships that could be construed as a potential conflict of interest.

## Supplementary Material

Supplementary Figure 1 Clinical features of cutaneous leishmaniasis patients and healthy controls participating in the study. NA, Not applicable.

Supplementary Figure 2 Proliferative capacity evaluated by Ki67 staining of CD4^+^ and CD8^+^ subsets [Naïve; Central memory (CM) and Effector memory (EF)] from HC or CL stimulated with 0.5 μg/mL of anti-CD3 for 72 h. The graphs show the mean ± SEM. *P*-values were calculated using repeated measures ANOVA with the Tukey correction used for *post-hoc* testing. ^^**^^*p* < 0.01, ^^****^^*p* < 0.0001.

Supplementary Figure 3 Correlation between cytokines levels and frequency of CD4^+^ EMRA subset in HC (○) or CL patients (■) were tested using Pearson's correlation test. For all tests, a *p* < 0.05 was considered statistically significant.

Supplementary Figure 4 Inflammatory profile correlates with differentiation state. Correlation between cytokines levels and frequency of CD4 **(A)** and CD8 **(B)** subsets in HC or CL patients were tested using Pearson's 1124 correlation test. For all tests, a *p* < 0.05 was considered statistically significant.

Supplementary Figure 5 Characterization of CLA expression in T cells during cutaneous leishmaniasis. CD4^+^ and CD8^+^ T cells isolated from healthy controls (HC) (*n* = 12) and patients with active cutaneous leishmaniasis patients (CL) (*n* = 14) were stained for cutaneous leucocyte-associated antigen (CLA) and analyzed by flow cytometry. Representative cytometry plots and cumulative data of CLA- expressing cells. The graphs show the mean ± SEM. *P*-values were calculated using repeated-measures ANOVA with the Tukey correction used for *post-hoc* testing. ^^****^^*p* < 0.0001.
