# Supplementary figures and images for: Circulating Senescent T Cells Are Linked to Systemic Inflammation and Lesion Size During Human Cutaneous Leishmaniasis
Source: Front Immunol. 2019 Jan 4;9:3001. doi: 10.3389/fimmu.2018.03001 (PMC6328442; doi:10.3389/fimmu.2018.03001)

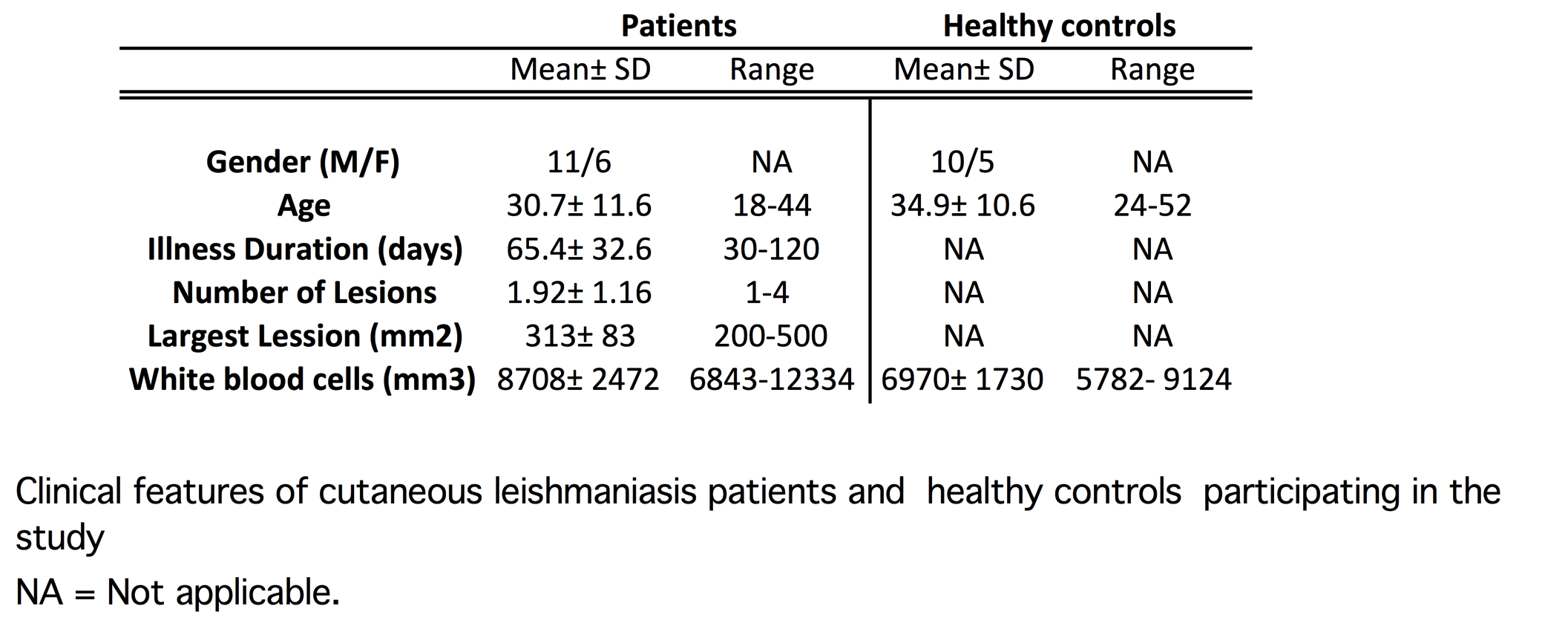

Supplement: Supplementary Figure 1 — Clinical features of cutaneous leishmaniasis patients and healthy controls participating in the study. NA, Not applicable. [file Image_1.TIFF]

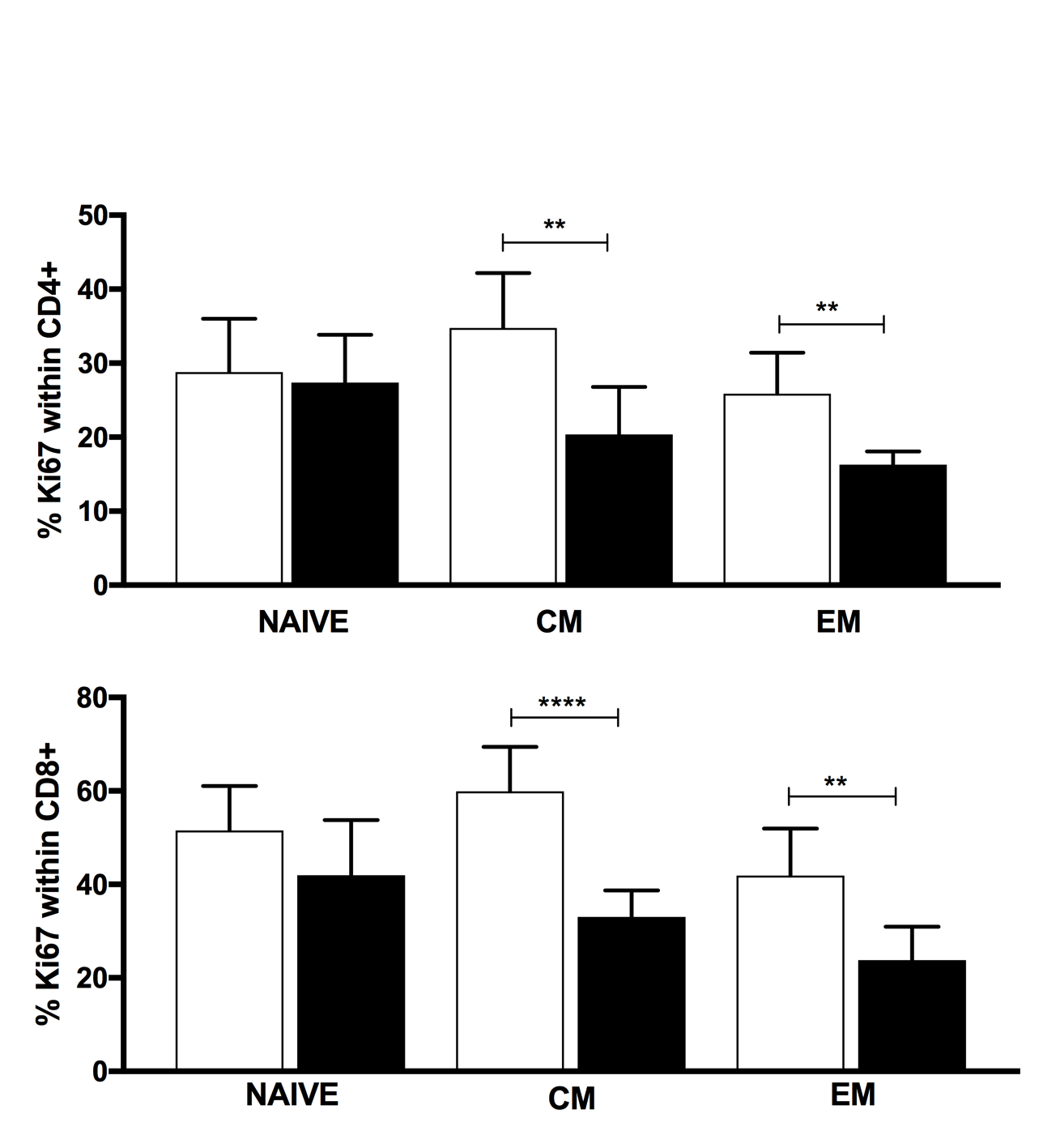

Supplement: Supplementary Figure 2 — Proliferative capacity evaluated by Ki67 staining of CD4+ and CD8+ subsets [Naïve; Central memory (CM) and Effector memory (EF)] from HC or CL stimulated with 0.5 μg/mL of anti-CD3 for 72 h. The graphs show the mean ± SEM. P-values were calculated using repeated measures ANOVA with the Tukey correction used for post-hoc testing. **p < 0.01, ****p < 0.0001. [file Image_2.TIFF]

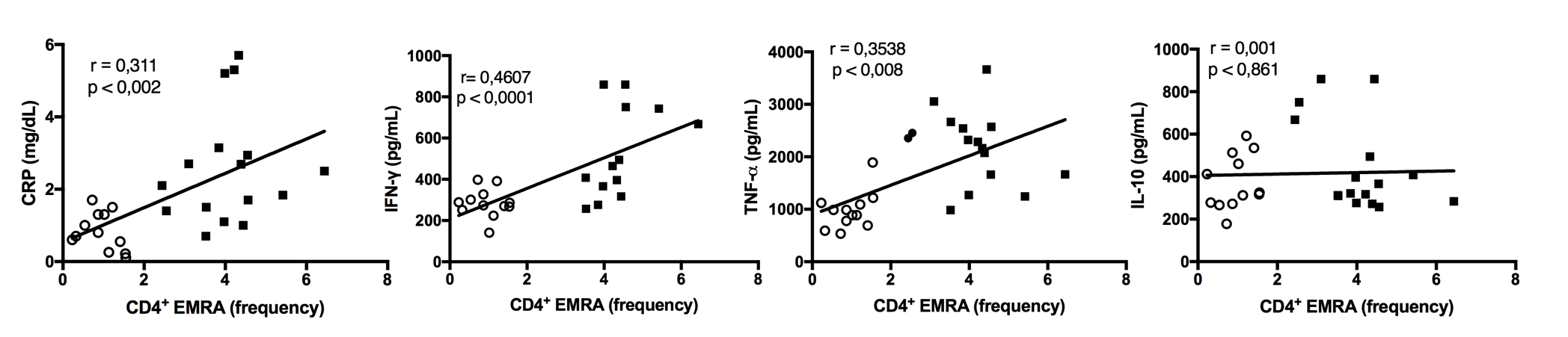

Supplement: Supplementary Figure 3 — Correlation between cytokines levels and frequency of CD4+ EMRA subset in HC (○) or CL patients (■) were tested using Pearson's correlation test. For all tests, a p < 0.05 was considered statistically significant. [file Image_3.TIFF]

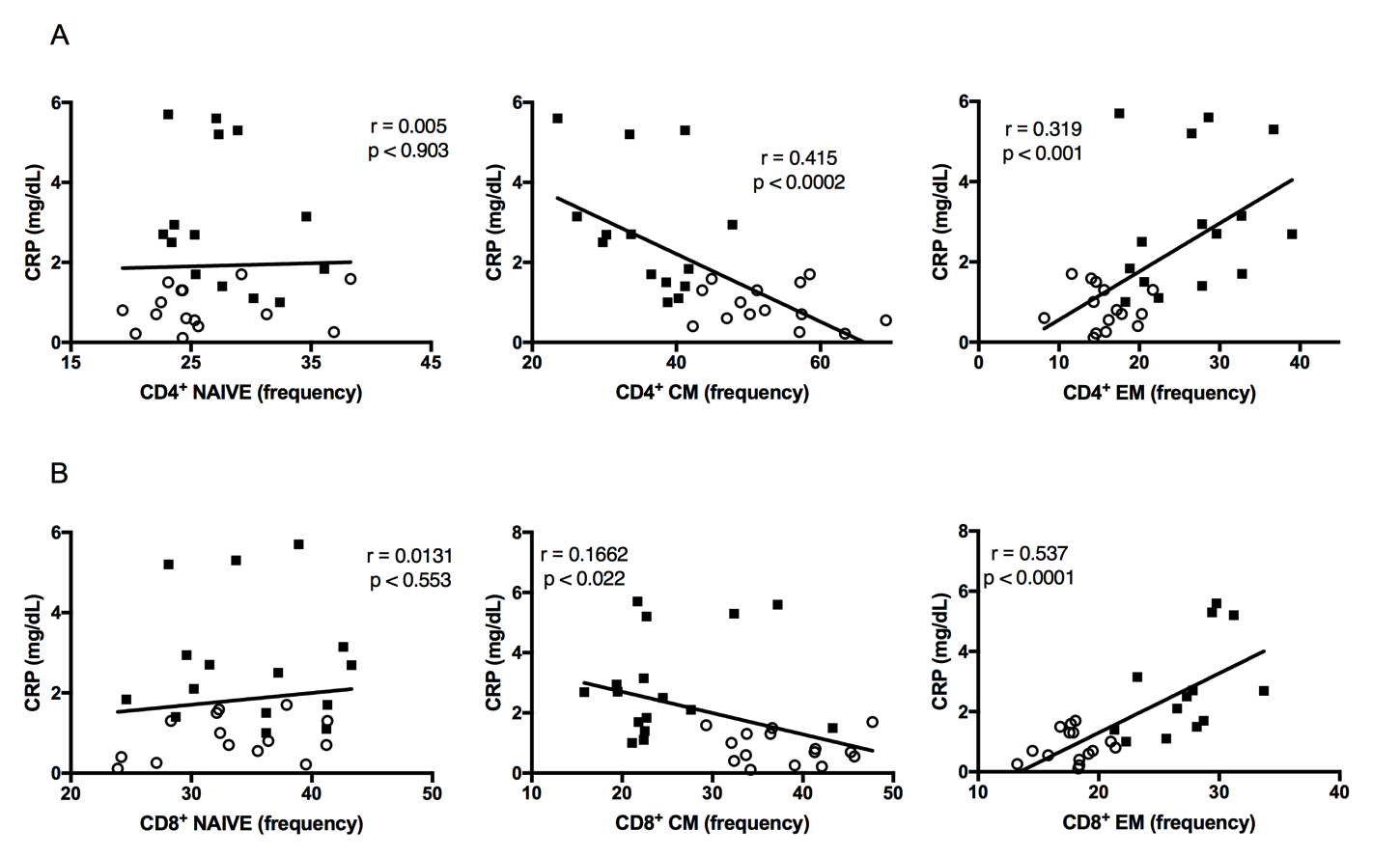

Supplement: Supplementary Figure 4 — Inflammatory profile correlates with differentiation state. Correlation between cytokines levels and frequency of CD4 (A) and CD8 (B) subsets in HC or CL patients were tested using Pearson's 1124 correlation test. For all tests, a p < 0.05 was considered statistically significant. [file Image_4.TIFF]

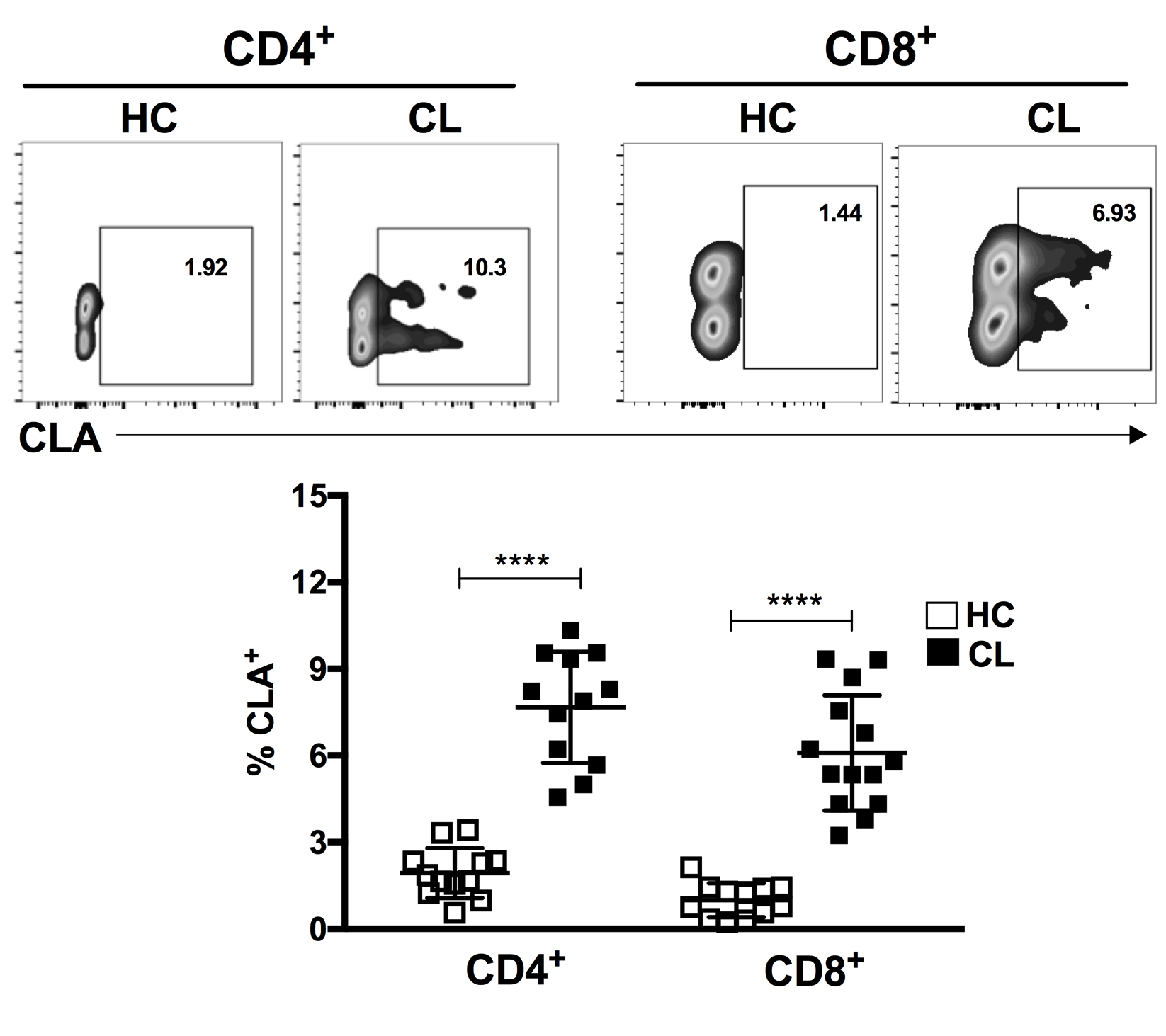

Supplement: Supplementary Figure 5 — Characterization of CLA expression in T cells during cutaneous leishmaniasis. CD4+ and CD8+ T cells isolated from healthy controls (HC) (n = 12) and patients with active cutaneous leishmaniasis patients (CL) (n = 14) were stained for cutaneous leucocyte-associated antigen (CLA) and analyzed by flow cytometry. Representative cytometry plots and cumulative data of CLA- expressing cells. The graphs show the mean ± SEM. P-values were calculated using repeated-measures ANOVA with the Tukey correction used for post-hoc testing. ****p < 0.0001. [file Image_5.TIFF]
